# Label‐free imaging of non‐deparaffinized sections of the human kidney to determine tissue quality and signatures of disease

**DOI:** 10.14814/phy2.15167

**Published:** 2022-02-08

**Authors:** Angela R. Sabo, Seth Winfree, Sharon B. Bledsoe, Carrie L. Phillips, James E. Lingeman, Michael T. Eadon, James C. Williams, Tarek M. El‐Achkar

**Affiliations:** ^1^ Department of Anatomy, Cell Biology, and Physiology Indiana University School of Medicine Indianapolis Indiana USA; ^2^ Department of Medicine Indiana University School of Medicine Indianapolis Indiana USA; ^3^ Department of Pathology and Microbiology Eppley Institute University of Nebraska Medical Center Omaha Nebraska USA; ^4^ Department of Pathology Indiana University School of Medicine Indianapolis Indiana USA; ^5^ Department of Urology Indiana University School of Medicine Indianapolis Indiana USA; ^6^ Indianapolis VA Medical Center Indianapolis Indiana USA

**Keywords:** histology, label‐free imaging, pathology

## Abstract

Label‐free fluorescence imaging of kidney sections can provide important morphological information, but its utility has not been tested in a histology processing workflow. We tested the feasibility of label‐free imaging of paraffin‐embedded sections without deparaffinization and its potential usefulness in generating actionable data. Kidney tissue specimens were obtained during percutaneous nephrolithotomy or via diagnostic needle biopsy. Unstained non‐deparaffinized sections were imaged using widefield fluorescence microscopy to capture endogenous fluorescence. Some samples were also imaged with confocal microscopy and multiphoton excitation to collect second harmonic generation (SHG) signal to obtain high‐quality autofluorescence images with optical sectioning. To adjudicate the label‐free signal, the samples or corresponding contiguous sections were subsequently deparaffinized and stained with Lillie's allochrome. Label‐free imaging allowed the recognition of various kidney structures and enabled morphological qualification for adequacy. SHG and confocal imaging yielded quantifiable high‐quality images for tissue collagens and revealed specific patterns in glomeruli and various tubules. Disease specimens from patients with diabetic kidney disease and focal segmental glomerulosclerosis showed distinctive signatures compared to specimens from healthy controls with normal kidney function. Quantitative cytometry could also be performed when DAPI is added in situ before imaging. These results show that label‐free imaging of non‐deparaffinized sections provides useful information about tissue quality that could be beneficial to nephropathologists by maximizing the use of scarce kidney tissue. This approach also provides quantifiable features that could inform on the biology of health and disease.

## Graphical abstract

Label‐free non‐deparaffinized sections provides useful information about tissue quality that could be beneficial to nephropathologists. This approach also provides quantifiable features that could inform on the biology of health and disease.

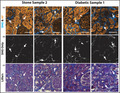

## 1 INTRODUCTION

The global prevalence of kidney disease exceeds 9% and is associated with significant morbidity, mortality, and economic burden (Carney, 2020). Our understanding of the pathogenesis of human kidney disease continues to evolve, in part due to advances in the molecular interrogation and imaging of human kidney biopsy specimens (Barwinska et al., 2021; El‐Achkar et al., 2021; Ferkowicz et al., 2021). Frequently, the specimens obtained after a diagnostic kidney biopsy may be too small or of limited quality and require a significant effort by nephropathologists to perform an appropriate diagnostic evaluation. Furthermore, based on recommendations from the College of American Pathologists, and to assure compliance with Clinical Laboratory Improvement Amendments (CLIA) laws and regulations, pathologists must retain paraffin blocks for a period of 10 years (Khoury et al., 2008). Therefore, the availability of kidney biopsy specimens for research purposes is limited. Endeavors such as the Kidney Precision Medicine Project are underway to study prospectively collected kidney biopsies with detailed clinical phenotypes (de Boer et al., 2021). These samples are acquired from altruistic donors and the available kidney tissue is both precious and scarce. Thus, an effective means to extract additional information from human biopsy specimens while conserving tissue is highly desirable and could benefit not only researchers but also patients who may be able to circumvent repeat biopsy procedures when the tissue obtained is limited.

In conventional diagnostic preparations, patient‐sourced biopsy specimens are fixed in 10% neutral buffered formalin (NBF), processed into paraffin blocks, and sectioned into delicate ribbons of tissue that are mounted on glass slides and subsequently deparaffinized for downstream histochemical and/or immunohistochemical techniques. On average, a diagnostic biopsy specimen from an 18G needle provides cylindrical tissue cores with a diameter of ~1 mm (Roth et al., 2013). From a specimen of this size, one can expect to obtain a limited number of histological sections of 2–5 µm thickness from the middle portion of the tissue, assuming the long axis of the tissue core is aligned parallel to the face of the paraffin block. Such specimens are typically prioritized for diagnostic use, thereby limiting the amount of tissue that can be released for institutionally approved research studies. To overcome this limitation, we believe there is value in developing tissue‐sparing techniques that both qualify and quantify histopathology. Such an approach would maximize the amount of information that could be obtained from a single specimen, which is especially important for tissue samples of limited or insufficient volume.

Label‐free fluorescence imaging describes the process of exciting and acquiring a fluorescence signal from tissue sections without the addition of any fluorescent probes. This process takes advantage of the endogenous fluorescence of several metabolites or proteins within the tissue (Hato et al., 2017). Furthermore, molecules with non‐centrosymmetric molecular structure such as fibrillar collagen (e.g., types I and II) and actomyosin produce a nonlinear optical effect known as second harmonic light when excited with an intense laser source (Chen et al., 2012; Strupler et al., 2007). Second harmonic generation (SHG) imaging has become more established for tissue‐based microscopy, and we have recently shown that combining label‐free and SHG imaging with subsequent multi‐fluorescence confocal microscopy in a single kidney section can be very valuable in evaluating the pathobiology of kidney disease (Ferkowicz et al., 2021). The use of label‐free imaging in conventional tissue processing and diagnostic preparations is not established (Bonsib & Reznicek, 1990). In particular, the application and utility of label‐free imaging in non‐deparaffinized human kidney sections have not been previously reported.

In this project, we sought to determine the feasibility and usefulness of label‐free imaging of formalin‐fixed paraffin‐embedded kidney sections without deparaffinization. We established a broadly applicable methodology that allows for pre‐qualification of the tissues to ensure that specimens of sufficient quality were selected for further analysis. Our results show that both structural and morphological information can be obtained from paraffin sections before deparaffinization. Furthermore, using additional specialized microscopy, we explored whether biologically relevant information, such as collagen content assessed by SHG imaging and changes in endogenous fluorescence, could be useful in defining a signature for disease, even before histological assessment occurs. This has been conducted previously in sections that have already undergone deparaffinization (Bhuiyan et al., 2021; Ranjit et al., 2015, 2016) but not in sections that are still embedded in paraffin. We propose that the knowledge from label‐free imaging of non‐deparaffinized sections could guide downstream processing in a conventional histologic workflow. Our findings also have implications for tissue economy in multimodal molecular and imaging interrogation of human kidney biopsy specimens.

## 2 METHODS

### 2.1 Sample collection

Tissue samples from patients with stone disease were obtained via percutaneous nephrolithotomy (Evan et al., 2003). Patients were randomly selected from an ongoing study in which all patients were consented for study (Indiana University Institutional Review Board protocol #1010002261). After biopsies were performed, samples were fixed with 4% paraformaldehyde (PFA) and then embedded in paraffin. All samples selected for this paper were from calcium oxalate stone formers with normal kidney function.

DKD and FSGS needle biopsy specimens were obtained from the Biopsy Biobank Cohort of Indiana (Indiana University Institutional Review Board protocol #1601431846; Eadon et al., 2020). These are diagnostic biopsies performed for clinical indication. After biopsy specimens were obtained, they were fixed with 10% formalin and then embedded in paraffin. The pathological diagnosis of DKD or FSGS was performed by a nephropathologist (C.P.).

### 2.2 Widefield epifluorescence microscopy

Non‐deparaffinized kidney tissue sections were imaged using a Keyence BX810 slide scanner and three different filter cubes, including DAPI, GFP, and TRITC. Images were collected using a Nikon PanFlour 10x/0.3 Ph1 air objective.

### 2.3 Second harmonic generation and two‐photon imaging

Non‐deparaffinized kidney tissue sections were also imaged with two‐photon microscopy using a 25x 0.95 NA Leica dipping objective with excitation provided by a MaiTai DeepSee tunable titanium‐sapphire laser (Spectra Physics) adjusted to 910 nm. The descanned pathway was configured for multiphoton imaging by fully opening the confocal pinhole and adjusting photomultiplier detectors to collect emissions from 440 to 460 nm (for second harmonic imaging, or SHG) and from 493 to 776 nm (for autofluorescence).

### 2.4 Single‐photon confocal imaging

Confocal imaging was conducted using a 25x 0.95 NA Leica dipping objective. Autofluorescence was collected in four different channels, ranging from 400 to 776 nm.

### 2.5 Staining sections with DAPI

Non‐deparaffinized kidney tissue sections were stained with DAPI to facilitate the calculation of the number of cells in the specimens. These sections were incubated with a 1:100 dilution of DAPI for 5 min, washed in PBS, and imaged as described above.

### 2.6 Histological staining

Deparaffinized tissue sections were stained with Lille's allochrome to facilitate the distinction of different collagen types in the tissue. Allochrome is similar to Masson's trichrome, except collagens within the tubule basement membranes and interstitial fibrosis stain purple and blue, respectively (Lillie, 1951).

### 2.7 Image analysis

Image analysis was conducted using the open‐source software ImageJ. The average fluorescence intensity of both the tubules and the glomeruli was measured in the three disease groups (stone, diabetes, and FSGS).

Cells were counted in images of DAPI‐stained sections with Volumetric Tissue Analysis and Exploration (VTEA), a software plugin for ImageJ (Winfree et al., 2017). VTEA utilizes nuclear staining, such as DAPI, to segment cells from the tissue volume for quantitation.

In order to calculate the amount of fibrosis in each sample, Otsu auto‐thresholding was applied to images with only the SHG channel included, in order to select for SHG signal with high intensity (Otsu, 1979). After applying the thresholding, the area occupied by the selected pixels was calculated using the “area fraction” measurement in ImageJ. This percentage was then used as the “percent area occupied by fibrillar collagen” in Figure 3d.

For correlation of SHG images to histology staining, three regions were randomly selected from four of the stone patient samples, for a total of 12 regions. Regions from the histology sections were scored by a nephropathologist as percent fibrosis. Regions from the SHG images were thresholded as described above, and the percent area occupied by SHG signal was calculated.

### 2.8 Statistical analysis

Statistical analysis was conducted using PRISM software. Mean ± standard deviation was reported. Statistical significance was determined using one‐way ANOVA and significance was set to *p* < 0.05.

## 3 RESULTS

### 3.1 Workflow for data extraction from fresh frozen paraffin‐embedded (FFPE) sections without deparaffinization

**FIGURE 1:**
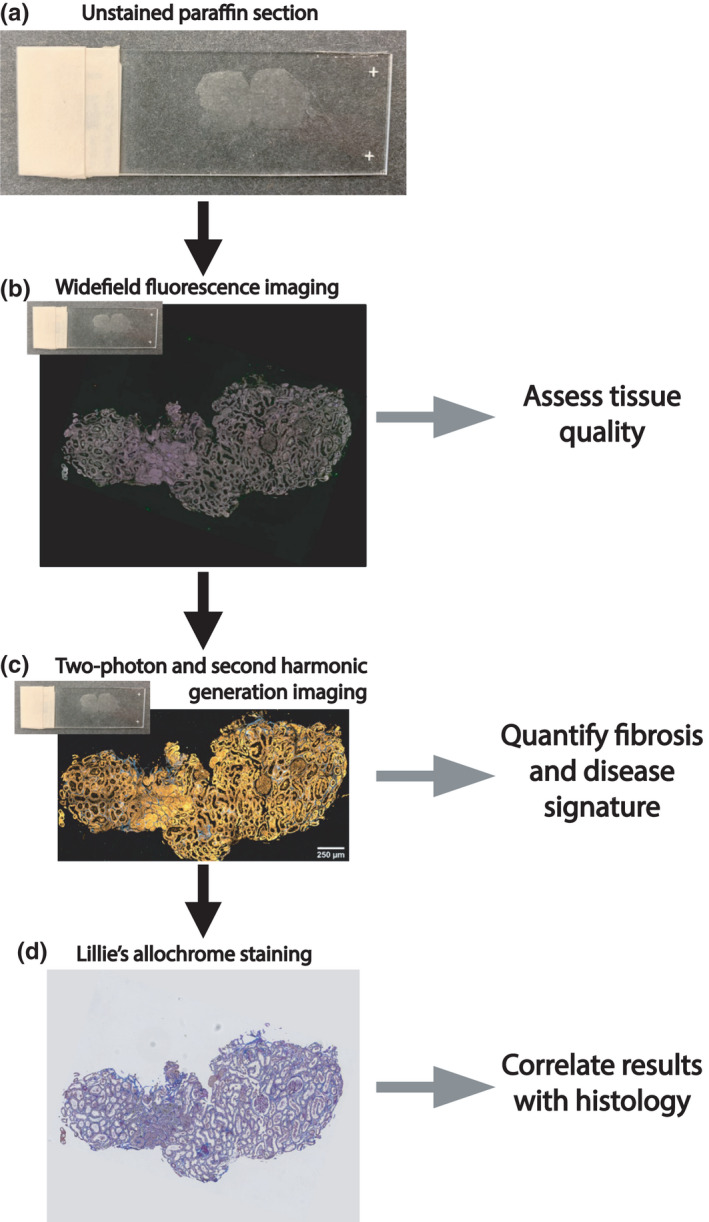
Flowchart representing the general methodology described in this paper. (a) Example of a non‐deparaffinized kidney tissue section. (b) Representative image of a non‐deparaffinized tissue section imaged using widefield epifluorescence. (c) The same section was imaged using two‐photon and second harmonic generation imaging. (d) This section was lastly deparaffinized and stained with Lillie's allochrome

Eighteen kidney biopsy specimens were used in this study and are described in Table 1. Specimens from the kidney cortex were either from kidney stone‐forming patients with normal kidney function obtained during percutaneous nephrolithotomy or from clinically indicated kidney biopsies of patients with an eventual primary pathology diagnosis of diabetic kidney disease (DKD) or focal segmental glomerulosclerosis (FSGS). After fixation, the specimens underwent standard processing for paraffin embedding and sectioning. The sections were imaged first using widefield epifluorescence microscopy to quickly assess the quality of the tissue section, followed by second harmonic generation imaging and endogenous fluorescence signature determination to allow for more quantitative methods of analysis to occur (Figure 1). Finally, for the purpose of adjudication, sections were deparaffinized and stained with Lillie's allochrome.

**TABLE 1:** Summary of tissue samples and the steps of the processing pipeline

Sample	Tissue obtained through	Clinical disease	No. of gloms	Steps in pipeline			
				Pre‐scanning	2P/SHG	Lillie's allochrome	Analysis
Stone 1	PCNL	Nephrolithiasis	2	X	X	X	
Stone 2	PCNL	Nephrolithiasis	5	X	X	X	X
Stone 3	PCNL	Nephrolithiasis	3	X	X	X	X
Stone 4	PCNL	Nephrolithiasis	16	X	X	X	X
Stone 5	PCNL	Nephrolithiasis	0	X			
Stone 6	PCNL	Nephrolithiasis	4	X			
Stone 7	PCNL	Nephrolithiasis	2	X			
Stone 8	PCNL	Nephrolithiasis	0	X			
Stone 9	PCNL	Nephrolithiasis	0	X			
Stone 10	PCNL	Nephrolithiasis	3	X			
Stone 11	PCNL	Nephrolithiasis	8	X			
Diabetic 1	PCNL	Nephrolithiasis, Diabetes	2	X	X	X	X
Diabetic 2	Diagnostic kidney biopsy	Diabetes	2	X	X	X	X
Diabetic 3	Diagnostic kidney biopsy	Diabetes	15	X	X	X	X
FSGS 1	Diagnostic kidney biopsy	FSGS	9	X	X	X	X
FSGA 2	Diagnostic kidney biopsy	FSGS	15	X	X	X	X
FSGS 3	Diagnostic kidney biopsy	FSGS	5	X	X	X	X
FSGS 4	Diagnostic kidney biopsy	FSGS	9	X	X	X	X

Abbreviations: 2P, two‐photon microscopy; FSGS, focal segmental glomerulosclerosis; PCNL, percutaneous nephrolithotomy; SHG, second harmonic generation.

### 3.2 Rapid tissue qualification using widefield epifluorescence microscopy

**FIGURE 2:**
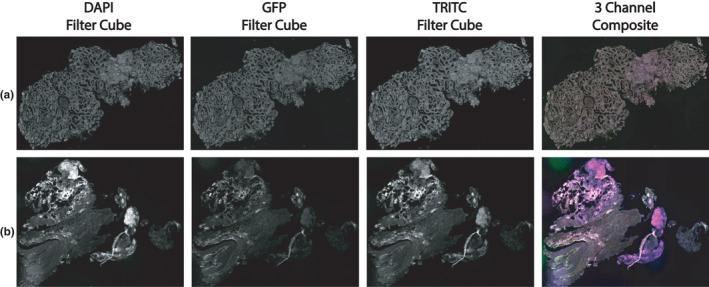
Widefield fluorescence imaging of non‐deparaffinized tissue sections allows for quality assessment. Imaging was performed using a fluorescence slide scanner with filter cubes set for DAPI, GFP, and TRITC fluorescent spectra. Two samples are shown above, with each channel displayed individually and as a composite of the three channels. The sample in (a) (stone patient sample 1) was used for the duration of the study, whereas the sample in (b) (stone patient sample 5) was not used due to poor sample quality

Widefield epifluorescence imaging was conducted on non‐deparaffinized tissue sections in order to provide a quick assessment of tissue quality without damaging the tissue or relying on time‐intensive analyses. In most cases, the total duration of this step did not exceed 10 min per tissue. To “pass” qualification, a section must have visible glomeruli (at least one must be clearly visible, but most times multiple glomeruli were observed), the tubules had to be morphologically distinguishable, and the tissue had to be of sufficient dimensions (qualitatively assessed as enough area to distinguish periglomerular space from other cortical areas) to provide an adequate area for analysis. In Figure 2, an example of a tissue that met all three requirements is shown (Figure 2a), as well as a specimen that did not successfully pass this step (Figure 2b). In total, 11 samples were chosen for further study: five specimens from stone‐forming patients (one of whom had also a history of diabetes), two with DKD, and four with FSGS (Table 1).

### 3.3 Quantifying fibrosis and endogenous fluorescence with label‐free imaging

**FIGURE 3:**
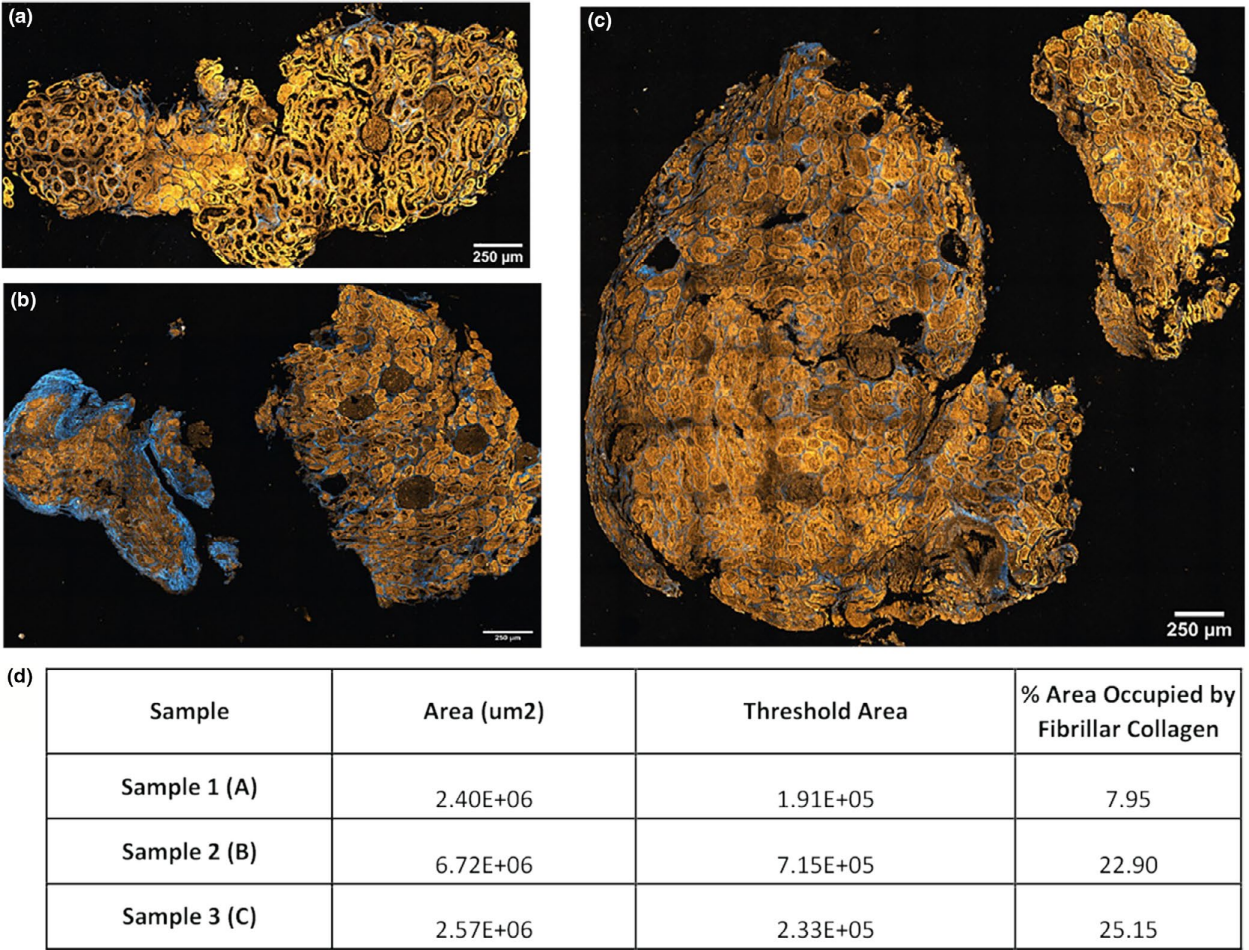
Two‐photon imaging and second harmonic generation imaging yield quantitation on collagen content. Two‐photon imaging and SHG imaging were conducted on the four non‐deparaffinized kidney tissue sections obtained from kidney biopsies of stone‐forming patients. (a–c) Tissue autofluorescence is displayed in orange, and collagens are shown in blue. To estimate the amount of collagenous content in each sample unsupervised thresholding on the signal was applied and was normalized to the tissue area. Results from that analysis are shown in (d)

To test if interstitial fibrosis could be imaged while a section was still embedded in paraffin, samples underwent SHG imaging. Such imaging was conducted by exciting the samples at 910 nm and collecting between 440 and 460 nm to select for fibrillar collagen. Endogenous fluorescence was captured concurrently using confocal microscopy. Figure 3 shows the SHG and autofluorescence images obtained from the nephrolithiasis kidney specimens. The amount of fibrosis present in the sample was measured by an unsupervised thresholding algorithm (Otsu, 1979) and was normalized to the total area of the tissue as reported in Figure 3d.

### 3.4 Histological adjudication of label‐free imaging

**FIGURE 4:**
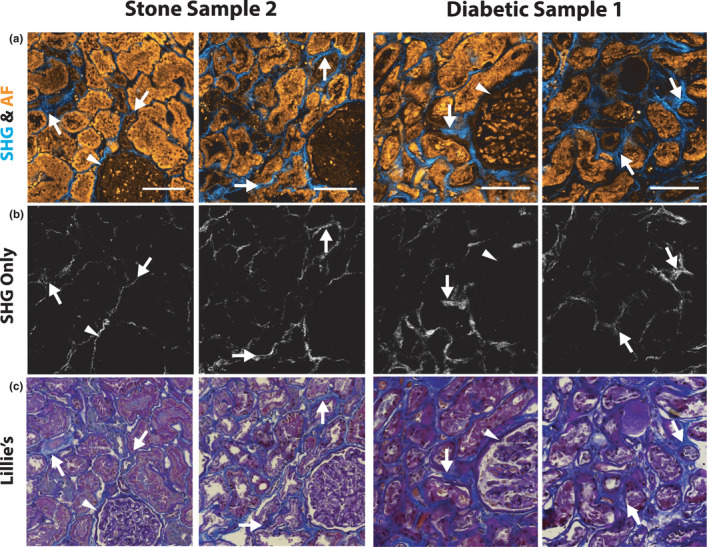
SHG imaging results correlate with Lillie's allochrome staining for collagens. (a) SHG imaging and two‐photon autofluorescence from two different samples. (b) The same images with only SHG signal displayed. (c) The same regions from the same sections are displayed after staining with Lillie's allochrome. The blue hue corresponds to fibrosis, dark purple is showing collagens that makeup the basement membranes (arrowheads, typically non‐fibrillar), and nuclei are stained dark brown/black. Arrows denote areas of signal in the SHG images that correlate with the Lillie's allochrome stain. Glomeruli and distal tubular segments have a dim autofluorescence compared to proximal tubules. Scale bars are 100 µm

Label‐free images collected from sections before deparaffinization were compared to images collected after deparaffinization and staining with Lillie's allochrome. Because this comparison is performed on the same sections without altering the orientation, the label‐free and stained images were spatially registered (Figure 4). Areas of high signal in the SHG channel showed a correlation with the blue collagen staining in the Lille's allochrome images, indicating areas of interstitial fibrosis. Furthermore, glomeruli and distal tubular segments had a unique dim autofluorescence signature compared to proximal convoluted tubules.

**FIGURE 5:**
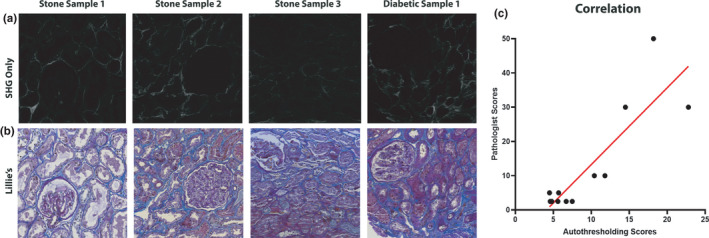
SHG imaging results correlate with Lillie's allochrome staining for collagens. (a) SHG signal of one region from each scored sample. (b) The same regions from the same sections after staining with Lillie's allochrome. (c) Shows the correlation of pathologist scoring of Lillie's allochrome staining to scoring from auto‐thresholding (*r* ^2^ = 0.752)

To quantify how well the SHG signal correlated with the Lillie's allochrome staining, regions were selected at random across four samples and scored. SHG images were “scored” using Otsu auto‐thresholding, as described above, and Lillie's allochrome images were scored by a nephropathologist. The correlation between the two sets of scores is shown in Figure 5c, along with representative images from the samples analyzed (Figure 5a,b).

### 3.5 Assessing changes in autofluorescence signature in disease

**FIGURE 6:**
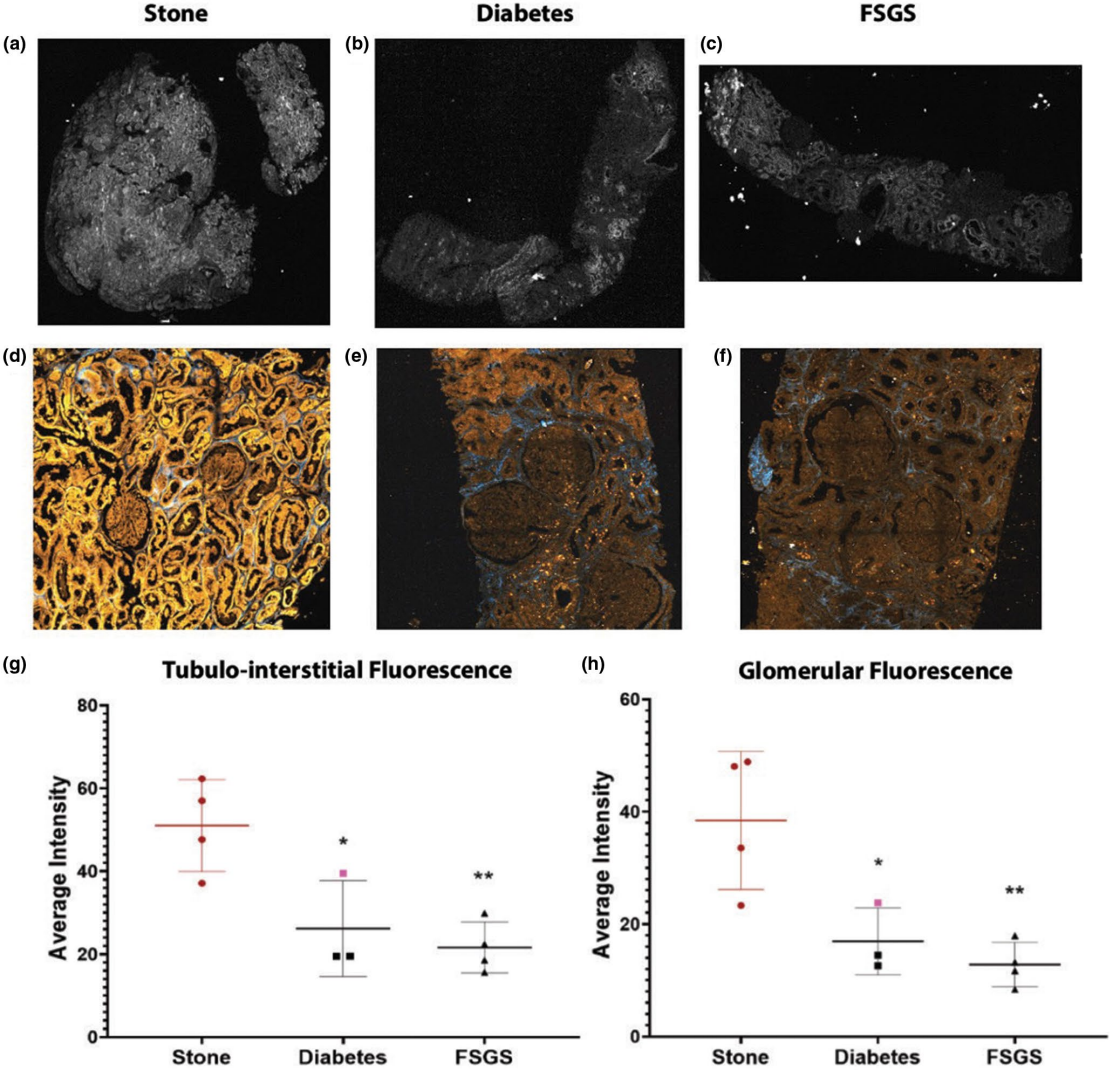
Detection of changes in tubulo‐interstitial and glomerular endogenous fluorescence during disease. (a–c) The analysis of tubulo‐interstitial endogenous fluorescence was conducted on images obtained in the GFP channel (epifluorescent imaging), as shown previously in Figure 2. (d–f) Glomerular analysis was conducted on images obtained using two‐photon and second harmonic generation imaging. (g, h) Show the results from the tubulo‐interstitial and glomerular analyses, respectively. (g) Data points represent the average intensity of the whole tissue. (h) Data points represent the average intensity of all of the gloms combined for each sample. The data point displayed in pink in the diabetes group is from a stone patient that also had history of diabetes. Single and double asterisks denote significant comparison between diabetes or FSGS to the stone reference group, respectively (*p* < 0.05). No difference was observed when comparing diabetes versus FSGS for either tubulo‐interstitial of glomerular endogenous fluorescence

The average autofluorescence signal from the tubulo‐interstitium was analyzed from images of the entire specimens obtained from widefield epifluorescence microscopy. An example image from each disease group is displayed in Figure 6. The tubulo‐interstitium from stone‐forming healthy patients had higher average endogenous fluorescence intensity compared to patients with diabetes or with FSGS (Figure 6d; *p* = 0.02 and <0.01, respectively). Similar findings were obtained from high‐resolution images of the glomeruli using two‐photon/SHG imaging (Figure 6e–h). Note that a stone‐forming patient who had diabetes was included in the diabetic group (Figure 6g,h).

### 3.6 Visualization of nuclei and measuring cell density in sections without deparaffinization

**FIGURE 7:**
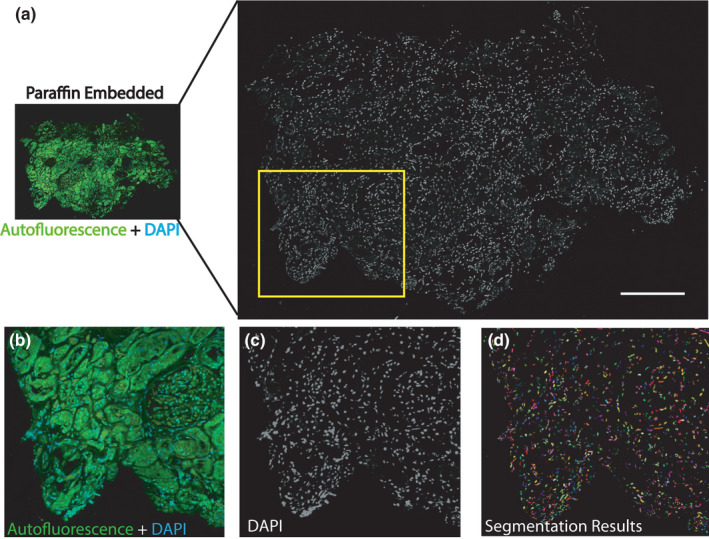
Nuclear staining and cytometry can be conducted on paraffin‐embedded samples without deparaffinization. (a) A non‐deparaffinized kidney section (10 µm thickness) was incubated with DAPI and subsequently imaged using confocal fluorescence microscopy. A z‐stack spanning the entire thickness was obtained (step size 1 µm). Scale bar = 250 µm. (b, c) High magnification region from the yellow box in (a). Scale bar = 150 µm. (d) Segmentation results from tissue cytometry VTEA analysis. Each color represents an individual nucleus that was segmented. Total count was 11,863 cells for the image volume from (a). (e, f) Glomerulus from (b) is enlarged and displayed. Scale bar = 100 µm. (g) Segmentation results from VTEA analysis. In total, 141 nuclei were identified in the glomerulus with a density of 5.5 × 10^−4^ cells/µm^3^

The ability to visualize nuclei in kidney tissue sections without deparaffinization was tested by applying DAPI to the paraffin‐embedded sections. Imaging for this experiment was conducted with confocal microscopy, the results of which are shown in Figure 7. Our results show that we could successfully label all the nuclei in a non‐deparaffinized tissue section. Such an approach allows the performance of tissue cytometry analysis on the tissue using the volumetric tissue exploration and analysis (VTEA) software (Winfree et al., 2017). There are numerous analyses that can be explored with the ability to label nuclei on non‐deparaffinized samples, ranging in complexity from cell count/cell density analysis to machine learning techniques to determine cell types based on the nuclear morphology, as we have previously shown with fresh frozen as well as 4% PFA‐fixed kidney specimens (Woloshuk et al., 2021). By segmenting all the nuclei in the imaging data, the total number of cells present in the tissue section shown in Figure 7a was measured and found to be 11,863 nuclei. Considering the autofluorescence signal of a specific structure, the cellular density in a specific region of interest (such as the glomeruli) can then be calculated (Figure 7). An example of this analysis is shown in Figure 7e–g, where we focus on a glomerulus. The results from the segmentation using VTEA are shown in Figure 7g, where we identified 141 cells in that particular glomerular cross‐section.

## 4 DISCUSSION

Our study demonstrates that a sizable amount of actionable information can be obtained from tissue sections while they are non‐deparaffinized. These data can be used for multiple applications such as qualification of the tissue for content and quality, quantitation of fibrosis, determination of disease signature or even nuclear staining without deparaffinization, and tissue cytometry analysis. Since paraffin embedding of tissues is a standard and common practice, maximizing the amount of information that can be obtained before committing the tissue to downstream analysis could have potential advantages for workflow efficiency and increasing tissue utility and extending usability. Furthermore, in the case of scarce tissue, the proposed approach has direct implication on tissue conservation.

Extracting information that could guide downstream use does not need to occur in highly specialized settings, because visualization of tissue content and quality could be performed with widefield epifluorescence microscope, an instrument that is widely available in most pathology labs. This commonly available technology can inform on the size of the tissue, glomerular content, and general tubule condition, without the need for staining or time‐intensive analysis. A pipeline for screening paraffin‐embedded tissue sections using widefield fluorescence could improve the efficiency of downstream processing. For example, an optimal diagnostic sample for glomerular diseases would include 15 or more glomeruli, and sometimes more than 20 glomeruli for diseases such as FSGS (Pritzker & Nieminen, 2019; Roth et al., 2013). By screening sections before staining, the optimal diagnostic sample could be identified a priori. Furthermore, a survey of the quality and content of all paraffin‐embedded sections could be very valuable for subsequent assignment to various assays and enhanced techniques (Messias et al., 2015).

Specialized high‐content data can also be obtained from paraffinized tissue sections, such as the measurement of interstitial fibrosis with SHG imaging or applying nuclear staining to perform tissue cytometry and measure cellularity in various structures. Such data could be used in conjunction with other downstream imaging and molecular assays to maximize the use of limited tissue, such as in the case of a kidney biopsy specimen. The specificity of endogenous fluorescence to the type of tubules and structures could be leveraged for use in future machine learning applications to delineate the content of a tissue section at high resolution (Liu et al., 2020; Rivenson et al., 2019). Furthermore, our preliminary data suggest that endogenous fluorescence itself may be altered by disease and could be potentially used for disease screening (Croce et al., 2010). This needs to be validated in a larger dataset.

This study has limitations predominantly related to the sample size, which precludes us from making generalizable conclusions without validation in a larger study. Although the samples from surgeries and needle biopsies were processed in a similar standard protocol, they were performed in different laboratories. While there could be variations in practices that contribute to the differences seen between reference and disease samples, both laboratories have an established track record of expertise and collaboration in tissue processing (Evan et al., 2014, 2015), which makes this possibility less likely. The goal of our study was to show the feasibility of the approach and its application, and our preliminary findings warrant additional investigations in a larger study. This limitation also translates to the feasibility of applying the proposed technique to other tissue types. While testing various tissues was outside the scope of this current study, we definitively showed that samples of renal cortex could be scanned to confirm the presence of cortical tissue, as well determine the number of glomeruli present in the section. Another limitation to this study is the decision to limit the samples used to those that contained cortical tissue. The fibrotic content could vary from the cortex to the medulla, which could require different imaging and/or analysis techniques to be utilized.

In conclusion, our results show that label‐free imaging of paraffin‐embedded sections without deparaffinization is easily implemented on common microscopes and provides useful information about tissue quality as well as quantifiable features that could potentially inform on the biology of health and disease. Additional high‐content data could be obtained with more specialized imaging, with possible implications on tissue economy in multimodal molecular and imaging interrogation of sparse human kidney biopsy specimens.

## CONFLICT OF INTEREST

The authors have no conflict of interest to declare.

## AUTHOR CONTRIBUTIONS

Research design: Angela R. Sabo, James C. Williams, and Tarek M. El‐Achkar. Sample collection: Michael T. Eadon, Carrie L. Phillips, James E. Lingeman, James C. Williams, and Tarek M. El‐Achkar. Performing experiment: Angela R. Sabo. Analysis: Angela R. Sabo, Seth Winfree, Carrie L. Phillips, James C. Williams, and Tarek M. El‐Achkar. Manuscript drafting and editing: All authors. Approval of final draft: All authors.
